# Clinical response to non-surgical periodontal treatment in patients with interleukin-6 and interleukin-10 polymorphisms 

**DOI:** 10.4317/medoral.21795

**Published:** 2017-06-18

**Authors:** Georgios S. Chatzopoulos, Aikaterini-Ellisavet Doufexi, Anastasia Kouvatsi

**Affiliations:** 1 Advanced Education Program in Periodontology, Department of Developmental and Surgical Sciences, University of Minnesota, USA; 2 Private practice limited to periodontics and implant dentistry, Greece; 3 Professor, Department of Genetics, Development and Molecular Biology, School of Biology, Faculty of Sciences, Aristotle University of Thessaloniki, Greece

## Abstract

**Background:**

Genetic polymorphisms are commonly associated with altered transcriptional activity and possibly make individuals more susceptible to periodontal disease development, increased disease severity and poor treatment outcome. The study aimed to determine the effect of Interleukin-6 (IL-6) -572 G/C (rs1800796) and IL-10 -592 C/A (rs1800872) polymorphisms on the outcomes of non-surgical periodontal therapy in a Caucasian population.

**Material and Methods:**

Sixty-eight patients with chronic periodontal disease were grouped according to their genotype: IL-6, IL-10, IL-6 and IL-10 susceptible (SCP) and non-susceptible (NSCP). All individuals were clinically evaluated at the first visit, and blood sample were collected from patients after checking the inclusion and exclusion criteria of the study. All patients received non-surgical periodontal therapy from a single-blinded periodontist. Clinical periodontal measurements were repeated 45 days after therapy.

**Results:**

This population mean aged 47.63 years included 52.2% females and 58.2% non-smokers. Following DNA separation and genotyping, 65.7% of patients were homozygous carriers of the IL-6 - 572G; 49.3% were carriers of the IL-10 -592A- allele (AA and CA genotypes); and 35.8% carried SCP genotypes for both polymorphisms. The clinical parameters after therapy were not associated with the genotype status. The multiple logistic regression analysis did not show any statistically significant association between the genotypes and the variables tested.

**Conclusions:**

Within the limitations of this longitudinal study, it can be suggested that IL-6 -572 G/C and IL-10 -592 C/A polymorphisms as well as their combination do not influence the outcome of nonsurgical periodontal therapy in Caucasian patients diagnosed with chronic periodontal disease.

** Key words:**Gene polymorphism, genetics, interleukins, periodontal disease, treatment outcome.

## Introduction

Periodontal disease is defined as a multifactorial inflammatory disease that is marked by destruction of the supporting tissues around teeth including periodontal ligament, cementum, alveolar bone and it is the major cause of tooth loss if left untreated (1). The role of microbial plaque in the onset of periodontal disease is primary and it results from interaction between host and microbial factors. Although there are some risk factors of periodontal disease that can be modified including smoking, diabetes mellitus, oral hygiene, excessive fat and bacterial accumulation, genetic predisposition is an unmodifiable factor of periodontal disease progression (2).

Although microbial plaque is the primary etiologic factor in periodontal disease, a subsequent study of 117 adult twin pairs showed that 50% of susceptibility to periodontal disease is credited to heredity or genetic factors. In this study by Michalowicz and colleagues, monozygotic twins were more similar than dizygotic twins for all clinical measures and a statistically significant genetic variance was found for both the severity and extent of disease (3). In a population studied by Kornman *et al.* a specific interleukin-1 (IL-1) gene polymorphism was linked to periodontal disease. Eighty-six percent of the patients diagnosed with severe chronic periodontal disease had either the IL-1 genotype or were smokers (4). In a recent meta-analysis of 53 studies deduced that chronic periodontitis is significantly associated with IL-1A -889 C/T and IL-1B +3954 C/T polymorphisms, whereas a weak association was also detected between IL-1B -511 T/C and chronic periodontal disease (5).

Meta-analyses have also confirmed a positive association between IL-6, IL-10 gene polymorphisms and chronic periodontitis (6-9). IL-6 is a pleiotropic cytokine and its gene located in 7p15-p21 chromosome. It was described as a key regulator in human immune system that exhibits pro-inflammatory and anti-inflammatory roles (10,11). IL-6 levels were found to be increased in inflamed periodontal tissues compared to healthy (12). In a study with postmenopausal Japanese women, it was revealed that IL-6 -572 G/C polymorphism may have an association between periodontitis and low truncal bone mineral density (13). Both meta-analyses that aimed to clarify the association between IL-6 -572 G/C polymorphism and periodontal disease susceptibility revealed a significant increased risk of chronic periodontitis in patients with GG genotype (6,7). In the most recent meta-analysis, the origin of the population was also investigated, disclosing the high susceptibility of Europeans to periodontitis (7).

IL-10 is an anti-inflammatory cytokine and its gene is located in 1q31-32 chromosome, regulating the inflammatory immune response (14). This cytokine may regulate tissue destruction. More specifically, the progression of experimental periodontal dis-eases found to be associated with the expression of innate immune cytokines: IL-10 was associated with an increased expression of tissue inhibitors of metalloproteinases (TIMP-1, -2 and -3) and osteoprotegerin (OPG), and with reduced expression of matrix metalloproteinases (MMPs) and the receptor activator of nuclear factor-kappaB ligand (RANKL) (15). The relationship between IL-10 -592 C/A gene polymorphism and chronic periodontitis was suggested by two meta-analyses (8,9). Both of them, that included almost the same studies, showed a significantly higher susceptibility to periodontitis in carriers of the A allele. Caucasians were also recognized to have a significant association with this susceptible phenotype (8,9).

Initial periodontal treatment includes oral hygiene instructions and non-surgical periodontal therapy aiming to diminish plaque accumulation as well as gingival inflammation, and it is also effective in gaining attachment level (16). This type of therapy has also found to be the best option for improving quality of life in adults with periodontal disease (17). The fundamental purpose of periodontal treatment is the long term preservation of natural teeth in healthy periodontal tissues. The identification of probable genetic polymorphisms associated with progression and development of periodontal diseases may contribute in predicting the periodontal treatment outcome (18).

The effects of several susceptible genotypes to chronic periodontitis on the outcome of non-surgical periodontal treatment and tooth survival were examined in a meta-analysis (19). The limited number of the included studies as well as the lack of methodologically sound studies led to the conclusion that the clinical outcome as assessed by bleeding on probing, clinical attachment loss and plaque index, after non-surgical periodontal treatment was not associated with the genotype status of the patients with chronic periodontitis. In the first three months and in the long-term outcome, the probing depth was related with the genotype profile. However, the authors concluded that more publications are needed in the future to identify such associations (19).

The initial response of the host to the microbial invasion is to recruit cells through the production of cytokines (20). IL-6 is expressed in situations involving immune responses and inflammatory reactions such as infection and trauma by macrophages, neutrophils, keratinocytes, fibroblasts and endothelial cells (21). IL-6 stimulates the production of antibodies and acute-phase proteins, activation of T- and B-cell differentiation as well as it induces osteoclast differentiation (22). IL-10 is an important an-ti-inflammatory cytokine with significant immunoregulatory role. IL-10 is expressed by B- and T- cells, mast cells, eosinophils, macrophages and dendritic cells (23). Both IL-6 and IL-10 play a pivotal role in the pathogenesis of periodontal disease and elevated levels of these cytokines have been detected in the gingival crevicular fluid of periodontally inflamed tissues, while both cytokine levels have been decreased in the gingival crevicular fluid after periodontal treatment (24-26). The single nucleotide polymorphism (SNPs) in the promoter region of IL-10 gene -592 is associated with altered IL-10 levels, while the carriers of the IL-6 gene -572 C allele is related to higher levels of IL-6 than GG subjects in the inflammatory state (27,28).

The aim of this study was to determine the effect of IL-6 -572 G/C (rs1800796) and IL-10 -592 C/A (rs1800872) polymorphisms on the outcomes of non-surgical periodontal therapy (NSPT) in a Caucasian population.

## Material and Methods

- Selection of participants

Patients with chronic periodontitis were recruited from a private practice limited to periodontics and implant dentistry in Thessa-loniki, Greece over a period of 10 months, between September 2014 and June 2015. The purpose and the procedures of the study were completely analyzed, and all patients were given a written consent form in accordance with the Declaration of Helsinki. A total of 68 Caucasians from Greece (33 males, 35 females, mean aged 47.53±9.62 years) were included in the study. The included individuals were considered susceptible (SCP) or non-susceptible (NSCP) to chronic periodontitis, according to the IL-6 (-572 G/C) and IL-10 (-592 C/A) polymorphisms.

- Inclusion and exclusion criteria

Complete medical history and full-mouth periodontal examination were obtained from all individuals. All participants had to be in good general health, diagnosed with chronic periodontitis, aged between 30 and 70 years, with at least 16 teeth in the oral cavity. The participants had also to sign the consent form. The applied exclusion criteria were the following: history of periodontal therapy (non-surgical and surgical) within the past 12 months; history of severe medical disorders; history of systemic diseases; need for antibiotic prophylaxis; history of systemic antibiotic therapy in the past 3 months; pregnant or lactating females.

- Clinical measurements

All clinical measurements were conducted by a single, calibrated (weighted kappa index of agreement for pocket probing depths=0.88), and blinded to the genotype status of each patient periodontist (E.D). Probing pocket depth (PPD), clinical attachment loss (CAL), bleeding on probing (BOP), percentage of sites with PPD=4-6 mm, percentage of sites with PPD≥7 mm, percentage of sites with CAL=4-6 mm, and percentage of sites with CAL≥7 mm were recorded utilizing a standard manual periodontal probe (15 UNC probe, Hu-Friedy, Chicago, IL, USA). All clinical periodontal parameters were assessed at six sites around each tooth (mesio-buccal, mid-buccal, disto-buccal, mesio-lingual, mid-lingual, disto-lingual) for the whole mouth, except the third molars. Full-mouth measurements were obtained at baseline and 45 days after periodontal therapy. The cementoenamel junction, or any other anatomical landmark in case of restoration, was utilized as a reference for the examination of the CAL. The presence of BOP was evaluated 15 seconds after the periodontal probing.

The diagnosis of chronic periodontitis was based on the Armitage’s classification which was adopted by the American Academy of Periodontology (29). In details, 30% or less of sites with periodontal destruction describe a localized chronic periodontitis, whereas the diagnosis of generalized periodontitis requires at least 30% of sites with destruction. The severity of the disease is based on CAL: 1-2 mm (mild); 3-4 mm (moderate); ≥5 mm (severe) (29). Patients were considered to have chronic periodontitis when exhibited the following periodontal measurements: two or more non-adjacent site demonstrated PPD≥5 mm; CAL≥3 mm; BOP.

- Periodontal therapy

All patients enrolled in the study received oral hygiene instruction, supragingival prophylaxis, as well as full-mouth scaling and root planing (SRP) under local anesthesia. A single, calibrated, experienced and blinded periodontist (E.D) performed the periodontal therapy. Manual curettes (Hu-Friedy, Chicago, IL, USA) and ultrasonic instrumentation (KaVo SONOsoft LUX, Kavo, Germany) were utilized. Follow-up periodontal examination was repeated 45 days after non-surgical periodontal therapy by the same periodontist.

- Blood samples, DNA separation and genotyping

Genomic DNA was extracted from blood drops using a commercially available genomic DNA isolation kit (QIAamp, DNA mini blood kit, QIAGEN, Germany) according to the manufacturer’s instructions. Genotyping of the polymorphisms in IL-6 -572 G/C (rs1800796) and IL-10 -592 C/A (rs1800872) genes was performed by polymerase chain reaction, in a final volume of 25 ul with a standard protocol. The genotype of random samples was confirmed by sequencing analysis.

The IL-6 polymorphism genotypes were determined by using the 5’-GGAGACGCCTTGAAGTAACTGC-3’ and 5’- GAGTTTCCTCTGACTCCATCGCAG-3’ primers to generate a PCR product of 163bp that was then digested with BsrBI (NEB) restriction enzyme. The -572C allele lacks the digestion site, giving a fragment of 163bp, while the -572G allele gives two fragments of 102 and 61 bp.

Primer pair 5’-CAACTTCTTCCACCCCATCTTT-3’ and 5’-GTGGGCTAAATATCCTCAAAGTT-3’ was used to amplify a 477 bp sequence containing the IL-10 polymorphism. Restriction enzyme RsaI (NEB) cleaved the -592C specific PCR product into four fragments of 311, 116, 42 and 8 bp and the -592A product into five fragments of 240, 116, 71, 42 and 8 bp.

For the IL-6 and IL-10 polymorphisms the digested products were separated on polyacrylamide gels stained with silver nitrate.

- Groups of patients according to genotypes:

1) IL-6 group

• SCP subgroup: individuals susceptible to chronic periodontitis carrying the susceptible genotype: GG (susceptible allele G).

• NSCP subgroup: individuals non-susceptible to chronic periodontitis carrying the non-susceptible genotypes: GC or CC.

2) IL-10 group

• SCP subgroup: individuals susceptible to chronic periodontitis carrying the susceptible genotypes: CA or AA (susceptible allele A).

• NSCP subgroup: individuals non-susceptible to chronic periodontitis carrying the non-susceptible genotype: CC.

3) IL-6 and IL-10 group

• SCP subgroup: individuals susceptible to chronic periodontitis carrying both the susceptible genotypes for IL-6 and IL-10: IL-6 GG and IL-10 CA or AA.

• NSCP subgroup: individuals non-susceptible to chronic periodontitis carrying one or all three non-susceptible genotypes: IL-6 GC, IL-6 CC, IL-10 CC.

- Sample size calculation

The aim of the study was to assess the clinical outcome of the non-surgical periodontal therapy in individuals susceptible and non-susceptible to chronic periodontitis. The primary outcome variable was the PPD. A sample size calculation was performed before starting the study. A minimum difference of 1 mm in the mean full-mouth PPD values of each patient and a standard deviation (SD) of 0.5 mm was considered for the sample size calculation. Aiming to achieve a 95% power of the study, it was determined that 6 subjects per group would be essential. Thus, the included number of patients in our study (68 patients) was sufficient enough to detect clinical differences (PS: Power and Sample Size Calculation version 3.1.2, 2014).

- Statistical analysis

The Shapiro-Wilk test was utilized to assess the normality of the distribution. Chi-square test and Mann-Whitney U test were utilized to examine differences in SCP and NSCP patients as far as age, gender, smoking status, alcohol consumption and number of teeth are concerned. Mann-Whitney and Wilcoxon tests were performed to evaluate the differences of the clinical measurements (PPD, CAL, BOP, percentage of sites with 4-6 mm and ≥7 mm PPD and CAL) between the SCP and NSCP groups at the same (baseline or 45 days after therapy) and different time points respectively (baseline and 45 days after therapy). Multiple logistic regression analysis was performed to analyze the association between the genotypes and the clinical and demographic variables. The Bonferroni test was also utilized for post-hoc analysis to adjust the results for the eight variables analyzed taking into consideration the multiple comparisons. All statistical procedures were performed with statistical software (SPSS v.19.0 for Windows, IBM, Armonk, NY, USA). Statistical significance was set at *P* <0.05.

## Results

This study included 68 Caucasians with chronic periodontal disease who were divided into two groups according to the presence or absence of the susceptible IL-6, IL-10 genotype: SCP and NSCP. One participant was excluded from the study as well as from the statistical analysis because he failed to come to the re-evaluation appointment due to change of residency. The final sample population of 67 Caucasians mean aged of 47.63 (±9.66) years was consisted of 35 (52.24%) females and 39 (58.21%) non-smokers. The IL-6 SCP and NSCP groups exhibited similar age and smoking history, whereas the male population showed significantly higher prevalence of periodontal disease susceptibility compared to the female population (*p*=0.04). In addition, the mean number of teeth of the SCP group was significantly higher in comparison to the NSCP group (*p*=0.01). Similar distribution was recorded in regards to age, gender, smoking status, alcohol consumption and number of teeth in the IL-10 group. The demographic characteristics of the study population are presented in  Table 1.

**Table 1:**
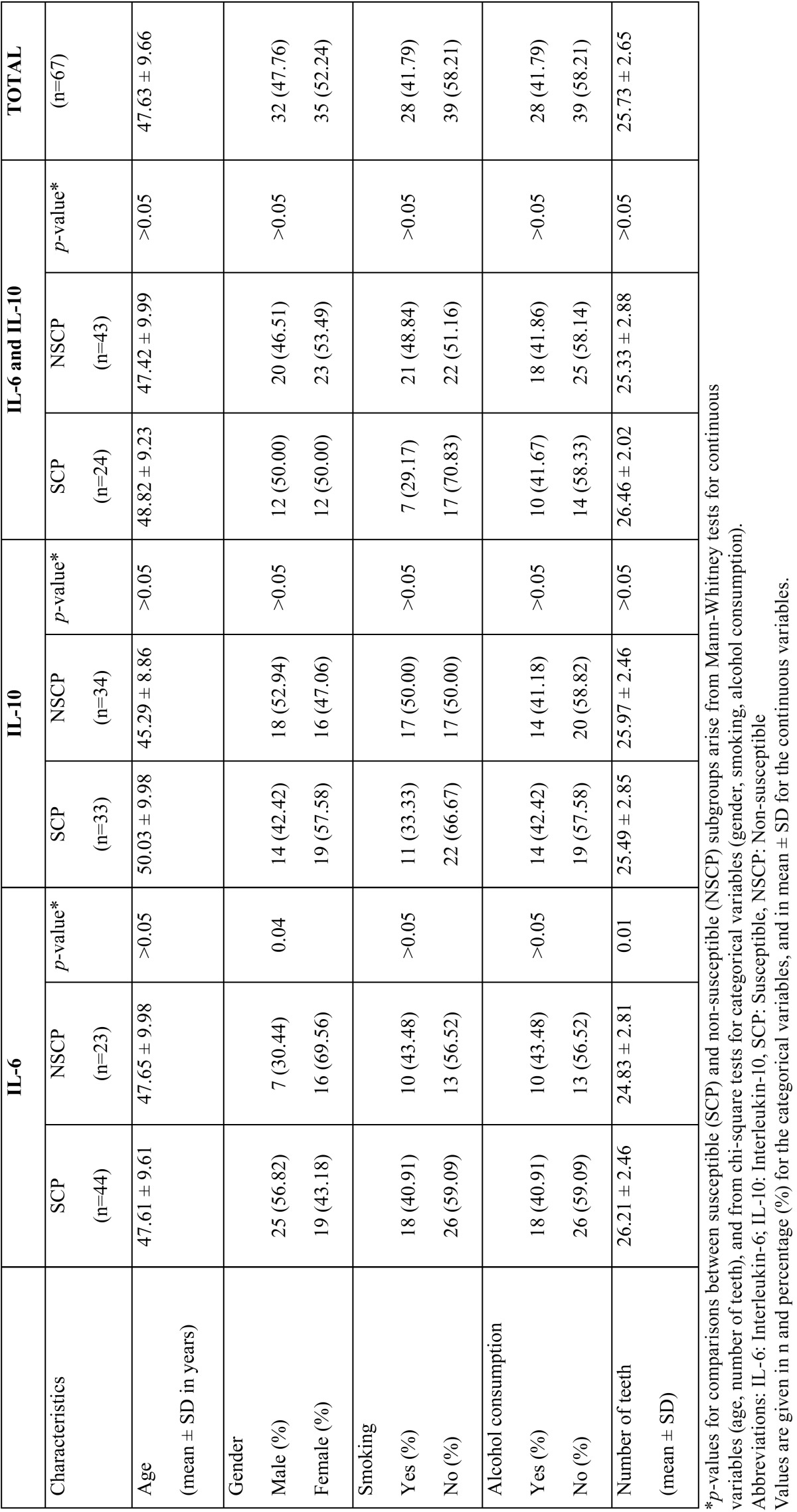
Demographic characteristics of the study population.

- Non-surgical periodontal treatment outcomes and genotype

The influence of genetic susceptibility to chronic periodontal disease in the IL-6 and IL-10 gene was evaluated. We analyzed the effects of non-surgical perio-dontal therapy on the clinical parameters between SCP and NSCP to chronic periodontitis, according to the IL-6 -572 G/C ( Table 2), IL-10 -592 C/A ( Table 3) polymorphisms as well as their combination ( Table 4). In particular, the differences in the clinical parameters between the SCP and NSCP groups were compared before and after non-surgical periodontal therapy. Both SCP and NSCP groups demonstrated similar clinical parameters (PPD, CAL, BOP, percentage of sites with PPD=4-6 mm, percentage of sites with PPD≥7 mm, percentage of sites with CAL=4-6 mm, and percentage of sites with CAL≥7 mm) at baseline (*p*>0.05), while 45 days after treatment all of them were significantly decreased (*p*<0.001). Furthermore, SCP and NSCP patients showed similar significant clinical improvements after periodontal treatment, but neither SCP nor NSCP groups revealed any significant difference in the clinical parameters at day 45.

**Table 2:**
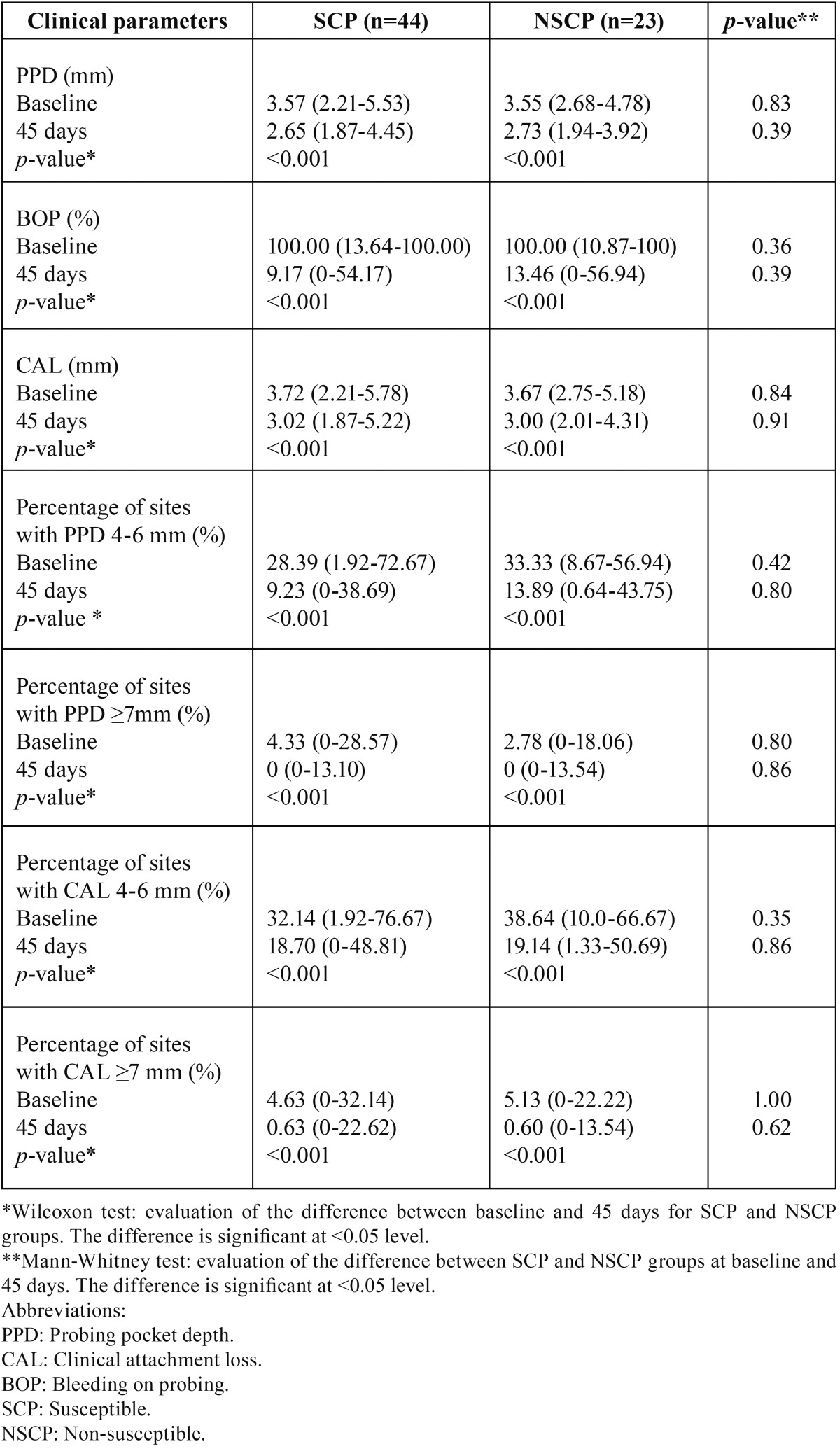
Clinical parameters (median; minimum-maximum) in chronic periodontitis patients at baseline and 45 days after non-surgical periodontal treatment of IL-6 susceptible (SCP) and non-susceptible (NSCP) patients.

**Table 3:**
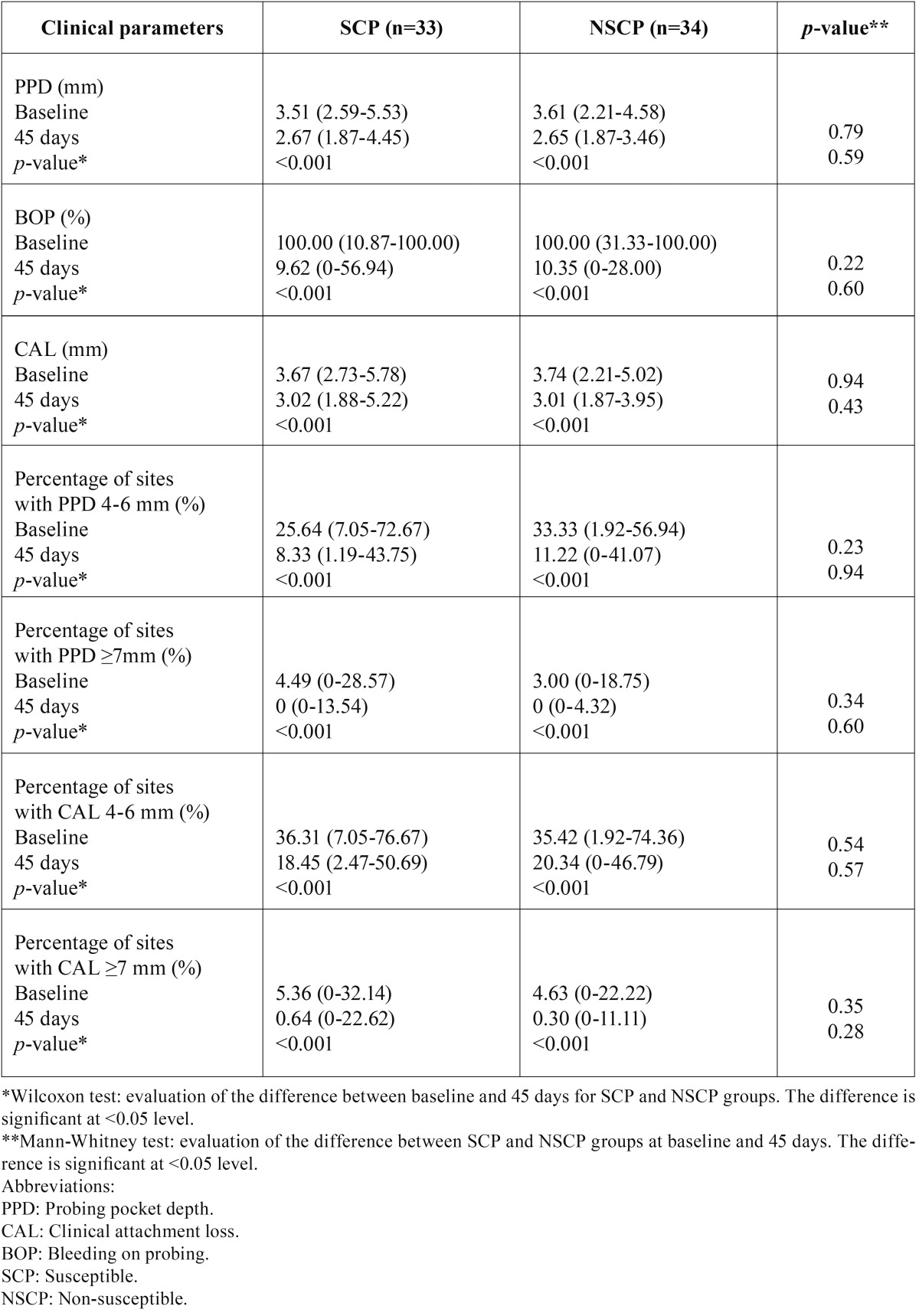
Clinical parameters (median; minimum-maximum) in chronic periodontitis patients at baseline and 45 days after non-surgical periodontal treatment of IL-10 susceptible (SCP) and non-susceptible (NSCP) patients.

**Table 4:**
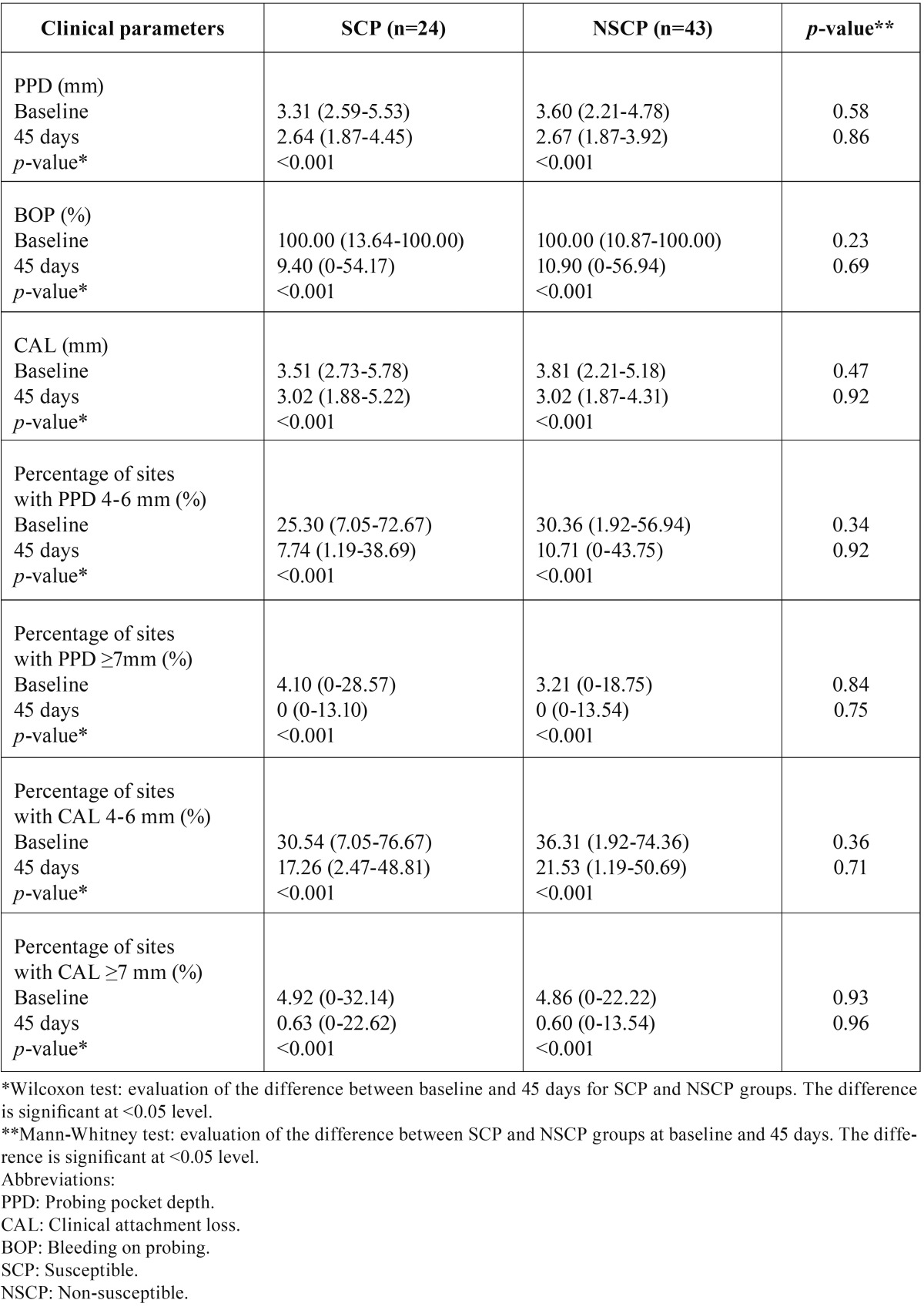
Clinical parameters (median; minimum-maximum) in chronic periodontitis patients at baseline and 45 days after non-surgical periodontal treatment of IL-6 and IL-10 susceptible (SCP) and non-susceptible (NSCP) patients.

- Multiple logistic regression analysis 

Multiple logistic regression analysis was utilized to identify whether there was any association between the examined genotypes and the demographic characteristics as well as the clinical parameters ( Table 5). At baseline, a significant association between IL-6 -572G/C, gender (*p*=0.01) and number of teeth (*p*=0.04) was detected. Similarly, 45 days after periodontal therapy, gender (*p*=0.01), number of teeth (*p*=0.01), probing pocket depth and clinical attachment loss (*p*=0.01) were significantly associated with this genotype. The results were adjusted according to the Bonferroni correction in order to avoid potential biased results from multiple comparisons, and only the association between probing pocket depth (*p*=0.04), clinical attachment loss (*p*=0.04) and IL-6 -572G/C remained. IL-10 -592 C/A did not exhibit any association with the examined clinical and demographic parameters.

**Table 5:**
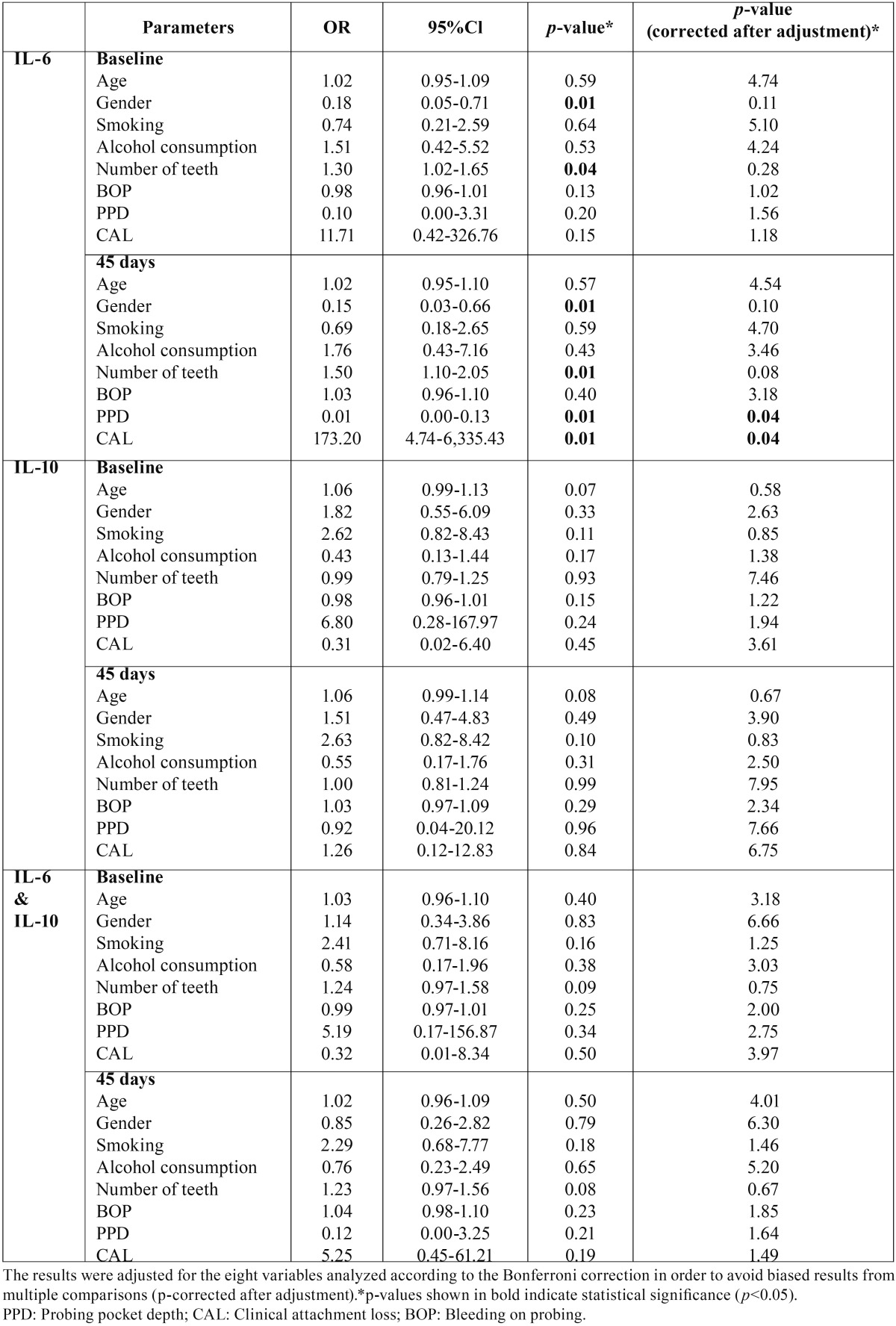
Multiple logistic regression analysis for the association between the IL-6 -572 G/C and IL-10 -592 C/A genotypes and the demographic and clinical variables at baseline and 45 days.

## Discussion

In this study, 67 patients of a private practice limited to Periodontics and Implant Dentistry were included and clinically examined. The effect of IL-6 -572 G/C and IL-10 -592 C/A polymorphisms on the non-surgical periodontal treatment outcome was evaluated. The hypothesis was that individuals susceptible to chronic periodontal disease would demonstrate decreased treatment response to non-surgical periodontal therapy compared to non-susceptible individuals. We failed to support our hypothesis, and all individuals showed similar significant clinical improvement. Individuals with chronic periodontal disease with similar periodontal clinical parameters at baseline, showed significant improvement in all clinical parameters 45 days after non-surgical periodontal therapy. However, none of the groups (SCP or NSCP) demonstrated any superiority. The results of our study suggest that IL-6 -572 G/C and IL-10 -592 C/A polymorphisms as well as their combination do not influence the periodontal treatment outcome.

According to a meta-analysis published by our group, this is the first study that evaluate the association between IL-6 -572 G/C, IL-10 -592 C/A polymorphisms and non-surgical periodontal treatment outcome (19). Both polymorphisms have been shown to be associated with chronic periodontal disease, as evidenced by various meta-analysis (6-9). Previous publications that aimed to assess the effect of genotype on the periodontal treatment outcome, examined polymorphisms of MMP-1, MMP-13, IL-1, IL-4, IL-6, IL-8 and mannose-binding lectin (MBL) (30-38). All studies were conducted in university settings and the included individuals were followed-up for 45 days to 6 months after non-surgical periodontal therapy.

The results of this study are in agreement with the majority of the publications. In a similar designed study from Corbi *et al.*, patients from a dental school with moderate and localized chronic periodontitis were examined by a single calibrated examiner before and 45 days after treatment. No significant differences were observed between IL-8 ATC/TTC haplotype and periodontal treatment outcome and the authors concluded that studies with patients from different ethnicities and diagnosed with generalized severe chronic periodontitis were necessary (36). In our study, European Caucasian patients from Greece with significantly more severe periodontal disease were included.

Similar studies demonstrated no significant differences between genetic polymorphisms and response to non-surgical periodontal therapy for MMP-13, IL-1, IL-4, and MBL (30-33,35,38). Pirhan and colleagues aimed to examine the effect of MMP-1 (-519 A/G; -1607 1G/2G) in a Turkish population with chronic periodontitis. Six months after periodontal therapy, the investigators reported that 519G allele carried higher percentage of sites with 4-6 mm clinical attachment loss after treatment. However, their finding did not show a clear relation to the progression of the disease, but further studies with different ethnic populations and larger samples were needed (34). In a prospective longitudinal study, D’ Aiuto *et al.* included 94 patients with generalized severe periodontitis and aimed to assess the relative contribution of several factors such as smoking, IL-6 polymorphisms, tooth mobility, tooth type and location on the treatment outcome after non surgical periodontal therapy. Six months after periodontal treatment, the authors detected a significant smaller decrease in the number of pockets in susceptible patients compared to non-susceptible ones (37).

IL-6 -572 G/C polymorphisms lead to an increase in the expression of IL-6, which plays a key role in the susceptibility to periodontal disease. The promoter region of the IL-6 controls the IL-6 transcription through a complex pathway, which is regulated by these polymorphisms (39). Similarly, IL-10 -592 C/A polymorphisms have been associated with an increased production of IL-10 protein, which is related with increased inflammatory response (9). However, our study did not detect any significant association between susceptible genotypes to periodontal disease and periodontal treatment outcome. The lack of association could be explained by other patient-related factors, which may impact significantly the outcome of non-surgical periodontal therapy. Poor compliance with oral hygiene and erratic compliance to maintenance are factors influencing the treatment outcome (40,41). Moreover, it is possible that combination of polymorphisms in more than two genes can have an effect on periodontal treatment outcomes. In our study, the short-term evaluation period and the demonstration of oral hygiene before the onset of therapy aimed to minimize these risk factors. Additionally, the presence of systemic conditions such as diabetes mellitus and smoking have also been shown to exhibit significant effect on periodontal healing after non-surgical therapy (42,43). For these reasons, individuals in good general health were only included in this study, while smokers were equally distributed among SCP and NSCP patients. In regards to the type of therapy, non-surgical periodontal therapy was provided and no surgical treatment or anti-infective therapy was performed in order to eliminate any possible effect on the treatment outcome.

The lack of socioeconomic information for the participants is one of the limitations of the study. However, the significant majority of the individuals treated in this private practice limited to Periodontics and Implant Dentistry were Greeks of middle class. Another limitation of the present study is the short-term follow-up. Although the participants of this study were clinically re-examined only 45 days after periodontal treatment, the study duration did not differ significantly from similar studies in the literature. The follow-up period of analogous studies ranged from 1 to 3 months. One of the strengths of this study is the big sample of a private practice population treated by a single periodontist leading to less operator bias. The sample size of this study was sufficient enough to detect clinical differences according to the sample size calculation which showed that 6 patients per group are essential to achieve a 95% power. The final size of the study population, 67 individuals, was able to reveal any potential association between the investigated genotypes and the treatment outcome.

Future studies are required to examine the potential association of different genetic polymorphisms of various genes with the response of the non-surgical periodontal therapy. Moreover, due to the multifactorial role of periodontal diseases, additional studies that will take into account a wide range of confounding factors such as smoking, ethnicities, races, bacterial strains and medical conditions are indicated. In the future we would like also to look into parameters that can have an epigenetic effect on polymorphisms. It is possible that different periopathogens or smoking or increased glycocylated hemoglobin can alter the genome and make the periodontal patient more susceptible to periodontal disease. Moreover, we can also make the hypothesis that smoking cessation or good oral hygiene or good glycemic control can have a beneficial epigenetic effect as far as the periodontal disease (44).

The analysis in the present study shows that after NSPT, genetic susceptibility to chronic periodontitis in the IL-6 and IL-10 genes did not affect the periodontal treatment outcome. IL-6 and IL-10 genes have both a significant effect on the initiation of periodontal disease and increased levels of these proteins have been found in the gingival crevicular fluid of sites with periodontal destruction (24,25). Following periodontal treatment, gingival crevicular fluid and serum IL-6 and IL-10 levels have been decreased or remained stable revealing that the resolution of the tissue inflammation and periodontal destruction is unlikely to significantly influence these protein levels (25,26,45-47). Moreover, it is possible that the expression of IL-6 and IL-10 can alter the levels of other cytokines or inflammatory factors that play a significant role only in the initiation of the inflammatory response and not in the healing process after treatment of periodontal disease. Although the association between IL-6 and IL-10 with periodontal disease initiation has been supported extensively in the literature, the findings of this study can confirm the lack of effect of IL-6 and IL-10 on the resolution of periodontal inflammation. In addition, albeit increased IL-10 levels have been associated with the presence of the SNP polymorphism in the promoter region of IL10 gene -592, this study fails to show a significant association between the presence of the polymorphism and the periodontal treatment outcome. This comes in agreement with previous data supporting the predominant anti-inflammatory role of this protein (14). However, no investigation was performed in the IL-10 protein levels that would shed some light in the association of the treatment outcome with the protein levels.

Although abundant evidence does support an association between IL-6 and IL-10 genes polymorphisms with susceptibility to chronic periodontitis, we observed that NSPT was not influenced by the susceptible genotypes. Treatment was equally effective for all studied parameters (probing depth, attachment loss, bleeding on probing, percentage of sites with 4-6 mm and ≥7 mm pocket depth and attachment loss). Further studies that will take into account a wide range of confounding factors such as smoking, ethnicities, bacterial strains and medical conditions are required to determine the potential association of different genetic polymorphisms of various genes with the response of the non-surgical periodontal therapy.
